# Connected diagnostics systems: The future of disease control in Africa

**DOI:** 10.4102/ajlm.v9i2.1365

**Published:** 2020-12-21

**Authors:** Noah Fongwen, Debi Boeras, Rosanna W. Peeling, Timothy Amukele

**Affiliations:** 1 International Diagnostics Centre, London School of Hygiene and Tropical Medicine, London, United Kingdom; 2 Global Health Impact Group, Atlanta, Georgia, United States; 3 Department of Pathology, Johns Hopkins School of Medicine, Johns Hopkins University, Baltimore, Maryland, United States

The coronavirus disease 2019 (COVID-19) pandemic has ushered us into a new era of global public health urgency, with diagnostics and laboratory medicine at its centre.^1^ To take advantage of this focus on diagnostics to leapfrog some of the barriers in low- and middle-income countries, we must first understand what is feasible and effective in our setting. This *African Journal of Laboratory Medicine’s* special issue on the ‘Future of Diagnostics’ offers us the opportunity to examine innovations in diagnostics that are already afoot in Africa. These innovations cover all three phases of testing and include a discussion of open (rather than closed) reagent systems for molecular testing, a tool for assessing the diagnostic capacity of a country, Nigeria’s experience establishing laboratory networks, tuberculosis data analytics, and mobile testing, reporting and surveillance – among others.

The global competition for COVID-19 reagents and molecular tests has taught us that African communities must strive for self-reliance; this self-reliance starts long before testing in the laboratory begins.^2^ Three articles in this issue address the need for changes outside the laboratory to support a better future for diagnostics. The article by Emperador et al. encourages the design and deployment of open reagent systems for molecular testing rather than the closed ones we have now.^3^ They also discuss some ways to address the changes in reimbursement and ownership that will be needed to support such a system.^3^ The article by Ondoa et al. presents the initial Africa-wide roll-out of a powerful new tool, the Joint External Evaluation Tool, which allows a country-wide comprehensive evaluation of diagnostic laboratory gaps and capabilities.^4^ Finally, the article by Naidoo and Ihekweazu describes how Nigeria built a national referral laboratory and established a country-wide specimen referral network to support national priority diseases including yellow fever, Lassa fever, monkeypox, cerebrospinal meningitis, cholera, influenza and other enteric pathogens.^5^ Networks of expert diagnostic laboratory sites for diseases of epidemic potential need to be established in a pan-African manner, so that specimens can be tested efficiently and reliably bio-banked to support local development and evaluation of new tests.^6^ The ability to develop and manufacture diagnostics in Africa is the future of disease control in Africa.^7^

The rapid spread of COVID-19 means that countries need real-time smart data systems for evidence-based decisions on public health measures and travel restrictions – as illustrated in three articles in this issue. First, Cassim et al. report on the use of an interactive dashboard at a high-volume laboratory that, when coupled with good management, dramatically improved the percentage of turn-around times within the expected range from 10% to 90%.^8,9^ Gous and Macek describe a new in-person and remote training programme, the TB Data Fellowship, which builds a tableau-based tuberculosis analytics capacity in low- and middle-income countries.^10^ Rapid advances in data connectivity and digital computing, coupled with increased access to internet coverage and portable communications such as mobile phones in Africa, provide us with a window of opportunity to adapt these technologies to strengthen diagnostic systems for the surveillance of diseases of epidemic potential, as well as for HIV, tuberculosis and neglected tropical diseases. A well-connected diagnostic system will cause a seismic shift in the way disease control is tackled in Africa.

Mobile phones and other mobile surveillance systems present some examples of changes in the status quo. In Senegal, epidemiological and diagnostic data on influenza-like illnesses from sentinel sites were collected and reported by phone through encrypted Short Message Service to the Ministry of Health.^11^ Peaks in the incidence of febrile syndromes were detected and the specific cause identified, leading to early notification of public health officials at the Ministry of Health.^11^ Fall et al. report in this issue on a successful pilot evaluation of the use of a mobile van equipped with a biosafety laboratory (complete with internal negative pressure chambers) that was deployed for outbreak investigations.^12^ In Tanzania, the control of neglected tropical diseases has taken a new leap forward by using mobile phones in early reporting of potential rabies cases, thus increasing compliance with post-exposure prophylaxis.^13^ In South Africa, researchers in the Africa Health Research Institute have developed a user-friendly mobile phone diagnostic test for HIV that uses a camera and an online app that allows for self-testing and interpretation of results.^14^ Whether this will translate to an increase in linkage to care is still being studied,^15^ but such an application can provide useful state-of-the art data on the transmission dynamics of HIV within communities, and has the potential to accelerate elimination efforts for HIV and other infectious diseases like syphilis and hepatitis. In Malawi, the Ministry of Health has partnered with Village Reach to establish a nation-wide health information hotline called Health Centre by Phone.^16^ Such innovation has been critical in disseminating useful health information and advice to the lowest level of the health system and dispelling myths and rumours during the COVID-19 pandemic.

This special issue has highlighted that Africa is a continent of qualified laboratory professionals and innovators. As new diagnostics are being developed on the continent, test developers should consider data connectivity as an essential component of any final product. Connectivity of new and existing point-of-care diagnostics should be fully functional and integrated with laboratory services to form connected diagnostic systems that will support disease surveillance and outbreak responses. Mobile phones are ubiquitous. We use them to access massive amounts of information and trust them with our mobile banking. They are powerful pocket computers and we should consider how we can use them to take healthcare to the last mile. The time to consider how we should harness this wealth of technology for the future of disease control in Africa is now.
